# Mobile-CEA – A Novel Surveillance Method for Patients with Colorectal Cancer

**DOI:** 10.1177/10732748221102780

**Published:** 2022-06-11

**Authors:** Sakari Pakarinen, Pirita Varpe, Anu Carpelan, Mari Koivisto, Heikki Huhtinen

**Affiliations:** 1 Department of Digestive Surgery, 60652 Turku University Hospital and University of Turku, Finland; 2 Department of Biostatistics, 8058 University of Turku, Finland

**Keywords:** colorectal cancer, surveillance, mobile phone, short message system, CEA

## Abstract

**Objective:**

The follow-up of the increasing number of cancer survivors threatens to overload the health care system. While short message system (SMS)–based communication is widely used in other areas of the health care system, there are no studies of its appliance in cancer surveillance. The aim of the current study was to analyze the acceptability, convenience and impact of a novel mobile phone messaging -based system (Mobile-CEA) on health personnel contacts in patients with colorectal cancer (CRC) during 2 years of follow-up.

**Methods:**

The follow-up data of 52 curatively treated patients with CRC (22 Mobile-CEA-, 30 standard surveillance) was collected retrospectively from the electronic archives. Mobile-CEA patient satisfaction was measured by a tailored non-validated questionnaire. Health personnel satisfaction was assessed by personal interviews.

**Results:**

Mobile-CEA surveillance group had less health personnel contacts than the standard surveillance group: median 3 (min 0–max 7) vs 5 (min 4–max 7) and 77.2% of the Mobile-CEA group had less than 4 contacts (minimum with the standard surveillance) to health personnel. There were no recurrences in either group. Mobile-CEA patients were satisfied with this novel follow-up method. Health personnel considered it as a practical and safe tool in CRC surveillance.

**Conclusion:**

Mobile-CEA surveillance seems to be a promising and effective follow-up method for curatively treated patients with CRC. Further studies and experiences are needed.

## Introduction

Colorectal cancer (CRC) is one of the most common cancers in the world. Its incidence rate is the third most common of all cancers and it is the second most common cause of cancer related mortality.^1^ Approximately 70 to 80% of patients with CRC present with potentially curable non-metastasized disease.^2^ After completion of treatment, most patients with CRC are followed up to detect recurrence, which could be treated curatively. The follow-up has traditionally been carried out by laboratory tests, computed tomography imaging, colonoscopy and physical examination. The ideal follow-up scheme for patients with CRC has not yet been settled, even though it has been assessed in many randomized studies.^3,4^.However, many national guidelines recommend follow-up every 3 or 6 months for 3–5 years focusing on the most effective combination of imaging modalities and laboratory measurements.^5-7^

The number of cancer survivors has increased over the last decade. Although the population is aging and the incidence of cancer is increasing, better cancer treatment and possibly earlier detection of the disease have resulted in improving survival.^8^ Providing follow-up for the growing number of cancer survivors will challenge the health care system, especially with the projected health care workforce shortages. Therefore, there is a need for new cost-effective follow-up methods.^8^ Mobile phone messaging has rapidly grown into a mode of communication with a wide range of applications, including communicating the results of medical investigations to patients. Alternative modes of communication of results include face-to-face communication, postal messages, calls, web-based health records, and email. Possible advantages of mobile phone messaging include convenience to both patients and health care providers, reduced waiting times for health services, and diminished health care costs.^9^ On the other hand dimensions like humaneness, personal touch and delivering understandable information may be reduced in telemedicine compared to in-person visits.^10,11^ A previous large study showed as high as a 94–99% patient satisfaction in telemedicine; 32% preferred it instead of in-person visits, 57% regarded it as good as traditional visit, 1% regarded it as worse than traditional visit and the rest were not sure.^12^ There are many available applications to offer health counseling but only few studies have evaluated the use of a mobile phone messaging for communicating results of medical investigations.^8^

It is estimated that among all subspecialities approximately 15% of practices use telemedicine for patient interaction as a substitute to in-person visits and 7.3% of practices use it in remote patient monitoring.^13^ In the previous literature, there are no studies concerning usage of telemedicine in cancer surveillance.

## Materials and Methods

### Mobile-CEA Surveillance System

Turku University Hospital has been offering a mobile phone messaging -based surveillance method (Mobile-CEA) as an alternative to standard surveillance for curatively treated patients with CRC since 2018. In the beginning Mobile-CEA surveillance was offered to patients with good prognosis (stage I-II) and typically after a few years of standard surveillance to find out if the Mobile-CEA method was functioning as expected and convenient to use. After positive feedback, patients were offered Mobile-CEA surveillance immediately after surgery, though still preferring lower stage diseases. Patients can voluntarily choose between Mobile-CEA and standard surveillance (Table 1). Cornerstone difference in these methods is that in the standard surveillance patients have appointed times with the health personnel for either visits or calls to review hemoglobin (P-Hb) and carcinoembryonic antigen (S-CEA) laboratory results. In the Mobile-CEA surveillance, aforementioned results are sent by an automated communicating short message system (SMS) directly to patients’ mobile phones.

**Table 1.** CRC Surveillance Scheme in Turku University Hospital.

	History and Physical examination	S-CEA and P-Hb	Colonoscopy	Body CT
Standard surveillance	every 6 month for the first 2 years, then annually for 3 years	every 6 months for the first 2 years then annually for 3 years	2–3 years after resection if preoperative was clear, then every 5 years	12 and 24 months in stage III-IV, then selectively
Mobile-CEA surveillance	Only patients with digitally examinable rectal anastomosis every 6 months for the first 2 years, then annually for 3 years	every 6 months for the first 2 years then annually for 3 years (mobile application)	2-3 years after resection if preoperative was clear,then every 5 years	12 and 24 months in stage III-IV, then selectively

Mobile-CEA service has been developed for streamlining and automating the interpretation of the laboratory results (P-Hb and S-CEA) by predefined criteria as well as for delivering these automatically as an SMS both to the patient and to the medical staff. At the start patients are informed of the SMS surveillance method and their suitability for using an SMS application is assessed. Reference values for S-CEA and P-Hb are set and they are adjustable on a case-by-case basis. The patient is given a long-term continuous laboratory referral by using the Weblab Clinical® application.

Short message system based laboratory control reminders are an essential part of the service. It is possible to determine how many days before the planned control time the patient will receive a reminder, and how many days after the planned control time a second reminder will be sent if the patient still has not visited the laboratory. Because a long-term follow-up may involve situations where the patient for some personal reason wants to visit the laboratory control even before the first reminder has been sent, the system automatically recognizes that there is no need for a reminder.

Laboratory results are reported to the patient in an SMS immediately after their completion. The system gives an alert to both the patient and medical staff if a patient misses a laboratory appointment or if the results are not within the reference range. These patients are always contacted by health personnel to inform them of the result and the need of any additional examinations.

Patients are informed of *“alarming symptoms,”* such as rectal bleeding, changed bowel function, and other progressive abdominal symptoms beforehand. They have a continuous possibility to contact medical staff by phone if they are worried about new symptoms or something in the surveillance is unclear. Patients can always revert Mobile-CEA surveillance to in-person visits if they want to.

### Patients and Follow-Up

This study consists of a total of 52 patients. The Mobile-CEA group included 22 patients who were consecutively entered to the Mobile-CEA surveillance system after curative surgery for CRC between May 2018 and February 2019. A comparative standard surveillance group (n = 30) was gathered from the hospital’s electronic health record system by identifying patients with CRC that had similar preoperative disease stage and were operated within the same time frame than the patients in the Mobile-CEA group. There were no significant clinical or statistical differences between these groups (Table 2). Patient data were collected retrospectively from the electronic archives with special interest in the number of visits and calls with the health personnel. The follow-up time was 2 years.

**Table 2.** Patient Characteristics.

Variable	Standard Surveillance (n 30)	Mobile-CEA Surveillance (n 22)	*p*
Age median (min-max)	73 (51.0–85.0)	70 (48.2–82.4)	.09^a^
Sex M/F (n)	11/19	10/12	.52^b^
ASA classification 1/2/3 (n)	1/11/18	0/9/13	1.0^c^
BMI median (min.-max)	26,0 (18.0–38.0)	26,0 (20.0–51.0)	.77^a^
Adjuvant chemotherapy (n)	1	2	.57^c^
Colon/rectum (n)	22/8	13/9	.28^b^
Stage I/II/III (n)	11/18/1	13/7/2	.11^c^
Preoperative CEA >4,6 ug/l (n)	10	7	.91^b^

^a^ Wilcoxon Two-Sample test.

^b^ Chi-Square.

^c^ Fischer's exact test.

Patients in the Mobile-CEA group were recruited for the patient satisfaction analysis by a phone call. All of them were willing to participate. A tailored non-validated questionnaire (supplementary material) was held during the call. In addition to the questionnaire, the patients’ free comments about the Mobile-CEA system were recorded. Health personnel satisfaction was assessed by interviewing nurses and surgeons that are accustomed to CRC surveillance at a regular basis.

### Statistical Analyses

Continuous variables are shown using medians and minimum and maximum values. Categorical variables are shown using frequencies and percentages. Differences in continuous background characteristics between study groups were studied using Wilcoxon two-sample test, with categorical background characteristics, chi-square test, or fisher’s exact test were used. All statistical tests were performed as 2-sided, with a significance level set at .05. The analyses were performed using SAS^®^ System, version 9.4 for Windows (SAS Institute Inc., Cary, NC, USA).

## Results

The Mobile-CEA surveillance group had less health personnel contacts than the standard surveillance group: median 3 (min 0-max 7) vs 5 (min 4-max 7), *P* < .0001. In the standard surveillance group the minimum number of contacts was 4 since patients had clinical appointments every 6 months for the first 2 years (Table 1). Every contact to gastrointestinal surgery department was accounted for, except visits related to stoma care. There are separate stoma nurses in gastrointestinal surgery department appointed only to stoma care and any other surveillance related issues are not addressed during those visits.

**Figure 1.**
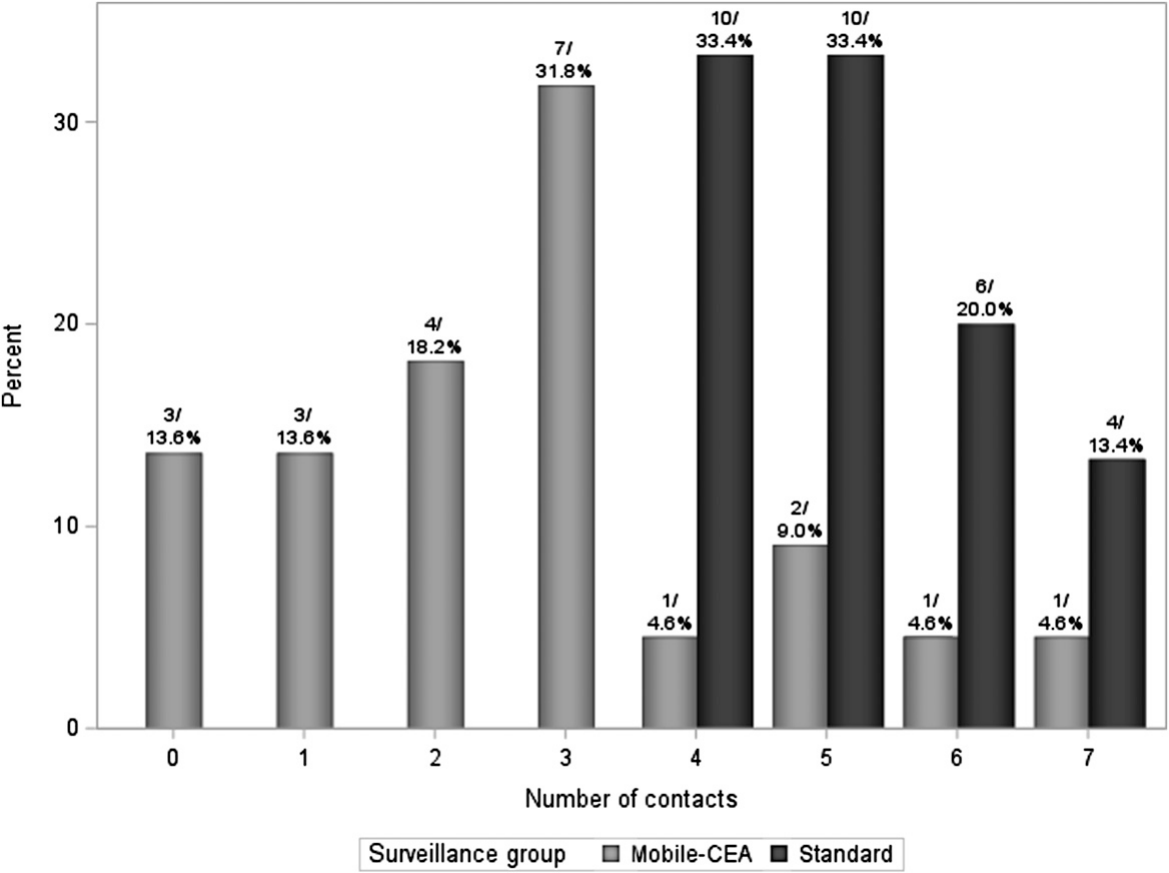
Number of contacts between the standard and Mobile-CEA groups (n/%) in 2-year surveillance.

**Figure 2.**
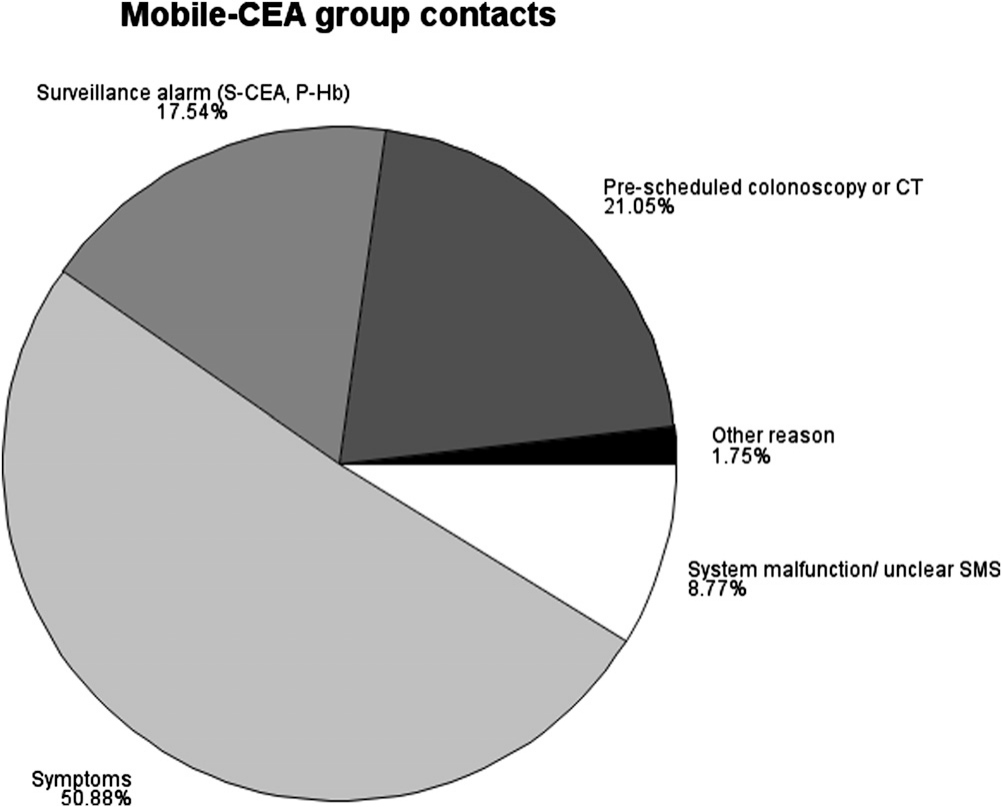
The reasons for contacts in the Mobile-CEA surveillance group.

In the Mobile-CEA surveillance group, 77.2% of patients had less than 4 contacts in the first 2 years (Figure 1). The reasons for contacts in the mobile-CEA group were the following: 12 (21%) pre-scheduled colonoscopies if the preoperative screening colonoscopy was incomplete or computed tomographies if patient had high risk disease (stage III), 29 (51%) contacts related to patient symptoms, 10 (17%) surveillance alarms when P-Hb or S-CEA levels were not within the reference range, 5 (9%) for system malfunction or unclear SMS message and 1 (2%) other undefined reason (Figure 2). There were no cancer recurrences in either group.

The patient satisfaction questionnaire was tailored in order to examine patient`s feelings and convenience with the Mobile-CEA follow-up. Everyone in the Mobile-CEA group answered the questionnaire (n = 22). Patients were satisfied in the Mobile-CEA surveillance method (Table 3). Two patients felt they could have had more information about the surveillance method and that they would have wanted more personal contact with the health personnel. In the free comments, patients mentioned the Mobile-CEA system to be convenient, flexible and easy to use. They were also pleased to receive results immediately after the completion of laboratory work i.e., without a need to wait for the appointed time from the medical staff. On the other hand, if the results were not in the reference range, it caused some stress to wait further instructions from the medical staff. None of the patients in the Mobile-CEA surveillance group were reverted to standard surveillance with in-person visits. Health personnel were also satisfied and found the Mobile-CEA system functioning as expected, although in the beginning there were some technical difficulties, and 5 contacts were due to excessive or unclear SMS-messages. After the beginning there have not been any further technical problems.

**Table 3.** Mobile-CEA Group Patient Satisfaction Questionnaire and Results (n = 22).

Question	Yes	No
Did you get enough information about the Mobile-CEA surveillance?	21 (95, 5%)	1 (4, 5%)
Are you satisfied in the Mobile-CEA surveillance method?	22 (100%)	
Did you get enough personal contact from the medical staff during surveillance?	20 (90, 9%)	2 (9, 1%)
Was it easy to reach personnel if you had any questions or problems during surveillance?	20 (90, 9%)	2 (9, 1%)

## Discussion

The Mobile-CEA is a promising and convenient CRC surveillance method. It appears to reduce contacts to health personnel and can therefore answer the need for a cost-effective postoperative follow-up method.^8^ In previous literature, there are only few cost-utility and cost-effectiveness studies assessing digital health systems and their results are controversial. Some cost-effectiveness studies demonstrate that telemedicine can reduce the costs, but not all. Among the main limitations of the economic evaluations of telemedicine systems are the lack of randomized control trials, small sample sizes, and the absence of quality data and appropriate measures.^14^ The Mobile-CEA automated surveillance system may reduce costs considerably, but further studies are needed. In the presence of the current COVID-19 pandemic, remote communication methods are preferred also in the health care systems in order to reduce unnecessary face-to-face contacts.^15,16^

The patients were satisfied with the Mobile-CEA surveillance system. This is in line with a recent review study showing high level of patient satisfaction in telemedicine mainly due to the convenience, decreased need of traveling and less in wait time for the results.^11^ Health personnel found the Mobile-CEA a useful and a safe way to observe patients with CRC. It is currently the primary surveillance system for CRC in Turku University Hospital. Transition from standard-to Mobile-CEA surveillance has had a significant diminishing impact on health personnel’s workload, which is always a consideration as the resources are limited.

In the beginning there were occasional technical difficulties and false alarms that caused some confusion to both patients and medical staff. Later, the system was improved so it allows to create individually adjusted reference rates for both S-CEA and P-Hb. This decreased the rate of false alarms that occurred with patients having some other medical condition that led to chronic anemia or hemoglobin level higher than the normal reference range.

There are several limitations in this study. The groups are small in this feasibility study and there were only 22 patients who were observed with the Mobile-CEA system straight after surgery. With the promising results of this study a larger study is planned in the future. The surveillance time in this study is also only 2 years. However, the differences in the number of contacts would likely be even greater in a standard 5-year surveillance. There is a selection bias in patients as the lower stage diseases are more present than the advanced ones. The patient satisfaction questionnaire was answered in a phone call with a surgeon at the end of the follow-up, which may have led to more favorable answers and recall bias. There is no validated Finnish patient satisfaction questionnaire which is why we had to use a tailored one. Therefore, our results on patient satisfaction are only descriptive. Considering that none of the patients had a recurrence during their short follow-up, it is likely that they are satisfied in the surveillance regardless of its method.

Most patients with CRC are elderly and operating with an SMS application does not suit for everyone, but it was found to be a surprisingly minor issue. A vast majority of elderly patients who had adequate functional and mental ability to be in postoperative surveillance to begin with, had also capability to use a mobile phone accordingly.

## Conclusion

The Mobile-CEA system was found a practical tool in CRC surveillance that also patients seemed to be satisfied with. Further studies and experience are needed to evaluate its long-term outcomes and cost-effectiveness in CRC surveillance.

## Supplemental Material

sj-pdf-1-ccx-10.1177_10732748221102780 – Supplemental Material for Mobile-CEA – A Novel Surveillance Method for Patients with Colorectal Cancer Click here for additional data file. Supplemental Material, sj-pdf-1-ccx-10.1177_10732748221102780 for Mobile-CEA – A Novel Surveillance Method for Patients with Colorectal Cancer by Sakari Pakarinen, Pirita Varpe, Anu Carpelan, Mari Koivisto and Heikki Huhtinen in Cancer Control
