# Supplementary material for: Mobile-CEA – A Novel Surveillance Method for Patients with Colorectal Cancer
Source: Cancer Control. 2022 Jun 11;29:10732748221102780. doi: 10.1177/10732748221102780 (PMC9209784; doi:10.1177/10732748221102780)
Supplement: sj-pdf-1-ccx-10.1177_10732748221102780 – Supplemental Material for Mobile-CEA – A Novel Surveillance Method for Patients with Colorectal Cancer [file sj-pdf-1-ccx-10.1177_10732748221102780.pdf]

Mobiili-CEA potilastyytyväisyyskysely

Potilaan nimi:

ID-nro:

1. Saitko riittävästi informaatiota Mobiili-CEA seurannasta? Kyllä/ Ei
2. Oletko tyytyväinen Mobiili-CEA seurantamuotoon? Kyllä/ Ei
3. Saitko riittävästi henkilökohtaista kontaktia hoitohenkilökunnalta seurannan aikana? Kyllä/Ei
4. Saitko helposti yhteyttä hoitohenkilökuntaan seurannan aikana ongelmien tai kysymyksien ilmetessä? Kyllä/Ei

Vapaat kommentit Mobiili-CEA seurannasta:
